# Supplementary material for: A Transcriptomic Approach to Elucidate the Mechanisms of Gefitinib-Induced Toxicity in Healthy Human Intestinal Organoids
Source: Int J Mol Sci. 2022 Feb 17;23(4):2213. doi: 10.3390/ijms23042213 (PMC8876167; doi:10.3390/ijms23042213)

Table S1. Input parameters for gefitinib human PBPK model in the Simcyp simulator and VIVD model [53] in the SIVA toolkit based on gefitinib properties and *in vitro* human intestinal organoid study design.

	Parameters and models	Gefitinib	Source
**Physiochemical properties**	Molecular weight (g/mol)	446.9	[63]
	Log P_{ow}	4.15	[29]
	Compound type	Diprotic Base	
	pK_a	5.4, 7.2	[28]
	Henry's Law Constant at 25 °C (Pa.m^3/mol)	1.02 x 10^{-13}	[64]
**Blood and plasma binding**	Blood-to-plasma ratio	0.8	[29]
	Alpha-1-acid glycoprotein equilibrium dissociation constant (μM)	8.8	[65]
	Human serum albumin equilibrium dissociation constant (μM)	54	[65]
	Fraction unbound in plasma	0.064	Calculated from equilibrium dissociation constants
**Absorption**	Absorption model	Advanced Dissolution, Absorption and Metabolism (ADAM) model	
	Unbound fraction of drug in gut enterocytes	1	Assumed
	Caco-2 passive permeability apical pH 7.4: basolateral pH 7.4 (10^{-6} cm/s)	10.41	[66]
	Human jejunum effective permeability (10^{-4} cm/s)	1.19	Simcyp scaled using Caco-2 passive permeability
**Distribution**	Distribution model	Full PBPK	
	Distribution prediction method	Rodgers and Rowland method [67,68]	
	Volume of distribution at steady state (L/kg)	24	[29]
	Kp scalar	0.96	Adjusted to match observed volume of distribution at steady state [29]
**Elimination**	Clearance type	Enzyme kinetics	
	CYP3A4 fraction metabolized	0.39	[69]
	Total *in vivo* clearance (L/h)	41.5	[29]
	Recombinant CYP3A4 *in vitro* intrinsic clearance (μL/min/pmol isoform)	1.24	Retrograde calculation
	Recombinant CYP2D6 maximum rate of metabolism (pmol/min/pmol isoform)	1.25	[70]

	Recombinant CYP2D6 Michaelis-Menten constant (μM)	6.94	[70]
	Recombinant CYP2D6 system	Baculovirus	[70]
	Additional human liver microsome intrinsic clearance (μL/min/mg protein)	290	Fitted to achieve total *in vivo* clearance [29]
***In vitro* organoid media parameters**	Culture media pH	7.4	Experiment
	Fraction unbound in foetal bovine serum	1	No foetal bovine serum used in media
	Volume of culture media per well (μL)	100	Experiment
***In vitro* intestinal organoid culture parameters**	Diameter of culture vessel well (mm)	6.4	Experiment
	Total volume of culture vessel well (μL)	360	Experiment
	Culture temperature (°C)	37	Experiment
	Average diameter of cell (μm)	5.32*	Experiment
	Number of cells per well	3000	Experiment

*Calculated based on average organoid area (4000 μm^2) and number of cells per organoid (180).

Table S2. Most relevant metabolic pathways (off-target) affected by gefitinib.

Name of the pathway	*Pathway source*	*Time (h)*	*Gefitinib concentration (μM)*	*q-value/number of DEGs*	
				**Colon**	**SI**
Glycolysis	Reactome	24	0.1	*NA*	**0.006/5**
			1	**7.82 x 10^{-5}/20**	**0.02/5**
			10	**5.0 x 10^{-4}/25**	**1.0 x 10^{-4}/9**
			30	**0.001/17**	**0.001/8**
		48	0.1	*NA*	*NA*
			1	**6.91 x 10^{-8}/13**	0.1/5
			10	**2.0 x 10^{-4}/18**	**0.02/6**
			30	**8.12 x 10^{-5}/19**	**0.02/7**
		72	0.1	*NA*	0.06/3
			1	**0.01/8**	**0.02/9**
			10	**0.006/19**	0.07/6
			30	**0.02/23**	**0.01/7**
TCA cycle	Reactome	24	0.1	*NA*	*NA*
			1	*NA*	*NA*
			10	*NA*	**0.01/9**
			30	*NA*	*NA*
		48	0.1	*NA*	*NA*

			1	*NA*	0.16/8
			10	*NA*	*NA*
			30	*NA*	*NA*
		72	0.1	**0.02/3**	*NA*
			1	*NA*	*NA*
			10	**0.03/33**	*NA*
			30	**1.02 x 10^{-17}/85**	*NA*
Pyruvate metabolism	KEGG	24	0.1	*NA*	0.08/2
			1	**0.04/7**	0.07/3
			10	0.07/6	0.07/3
			30	**0.004/11**	**0.04/4**
		48	0.1	*NA*	*NA*
			1	**0.01/8**	*NA*
			10	**0.03/9**	**0.04/4**
			30	**0.02/9**	**0.02/5**
		72	0.1	*NA*	0.08/2
			1	0.13/4	**0.002/8**
			10	**0.02/12**	0.1/4
			30	**0.02/15**	**7.0 x 10^{-4}/7**
Respiratory electron chain and ATP synthesis by chemiosmotic coupling, and heat production by uncoupling proteins	Reactome	24	0.1	*NA*	*NA*
			1	*NA*	*NA*
			10	*NA*	**0.05/6**
			30	*NA*	*NA*
		48	0.1	*NA*	*NA*
			1	*NA*	*NA*
			10	*NA*	*NA*
			30	*NA*	*NA*
		72	0.1	**0.009/3**	*NA*
			1	*NA*	*NA*
			10	0.11/22	*NA*
			30	**4.30 x 10^{-19}/70**	*NA*
Metabolism of lipids	Reactome	24	0.1	*NA*	*NA*
			1	**8.0 x 10^{-4}/61**	*NA*
			10	**0.003/68**	*NA*
			30	**0.008/73**	**1.23E-06/35**
		48	0.1	*NA*	**0.02/14**
			1	**5.0 x 10^{-4}/62**	*NA*
			10	**5.91 x 10^{-5}/86**	*NA*
			30	**3.0 x 10^{-4}/80**	**8.0 x 10^{-4}/35**
		72	0.1	*NA*	0.1/9
			1	**0.01/34**	*NA*
			10	0.09/95	*NA*
			30	**0.04/148**	**0.006/31**
Metabolism of amino acids	Reactome	24	0.1	*NA*	*NA*
			1	A	*NA*
			10	*NA*	**0.05/11**
			30	*NA*	*NA*
		48	0.1	*NA*	*NA*
			1	0.2/25	**7.0 x 10^{-4}/21**
			10	*NA*	**1.1 x 10^{-8}/27**

			30	*NA*	**0.02/18**
		72	0.1	**3.77×10^{-6}/8**	*NA*
			1	**2.29×10^{-13}/41**	**0.003/27**
			10	**1.70×10^{-14}/94**	**1.54×10^{-9}/33**
			30	**1.34×10^{-7}/133**	**0.004/20**
Metabolism of proteins	Reactome	24	0.1	*NA*	*NA*
			1	*NA*	*NA*
			10	*NA*	**0.01/45**
			30	*NA*	*NA*
		48	0.1	**0.006/5**	0.07/26
			1	*NA*	**1.0×10^{-4}/75**
			10	*NA*	**5.15×10^{-6}/70**
			30	*NA*	**0.004/74**
		72	0.1	**0.004/12**	*NA*
			1	**1.58×10^{-5}/95**	**3.0×10^{-4}/110**
			10	**1.42×10^{-15}/354**	**1.68×10^{-5}/86**
			30	**1.14×10^{-27}/566**	0.06/65
Cholesterol biosynthesis pathway	WikiPathways	24	0.1	NA	*NA*
			1	**7.33×10^{-10}/11**	*NA*
			10	**5.35×10^{-4}/7**	NA
			30	**0.03/5**	**2.89×10^{-15}/11**
		48	0.1	NA	NA
			1	**1.17×10^{-11}/12**	NA
			10	**2.24×10^{-11}/13**	NA
			30	**1.02×10^{-11}/9**	**6.36×10^{-10}/9**
		72	0.1	NA	NA
			1	**1.37×10^{-7}/8**	NA
			10	**3.50×10^{-4}/11**	NA
			30	**0.005/9**	**1.11×10^{-11}/10**
Drug metabolism by cytochrome P450	KEGG	24	0.1	*NA*	**0.02/4**
			1	**0.04/10**	**0.007/6**
			10	**0.02/12**	**0.007/6**
			30	0.08/11	**0.05/5**
		48	0.1	*NA*	**0.001/6**
			1	**0.04/11**	**0.05/6**
			10	**0.03/13**	**0.009/8**
			30	*NA*	**0.02/7**
		72	0.1	*NA*	**0.006/5**
			1	0.09/6	**0.04/8**
			10	*NA*	**0.005/8**
			30	*NA*	**0.004/8**

Legend: significant q-values < 0.05 (in bold) or not applicable (NA) when the respective pathways were not present; the number of genes does not mean significantly affected.

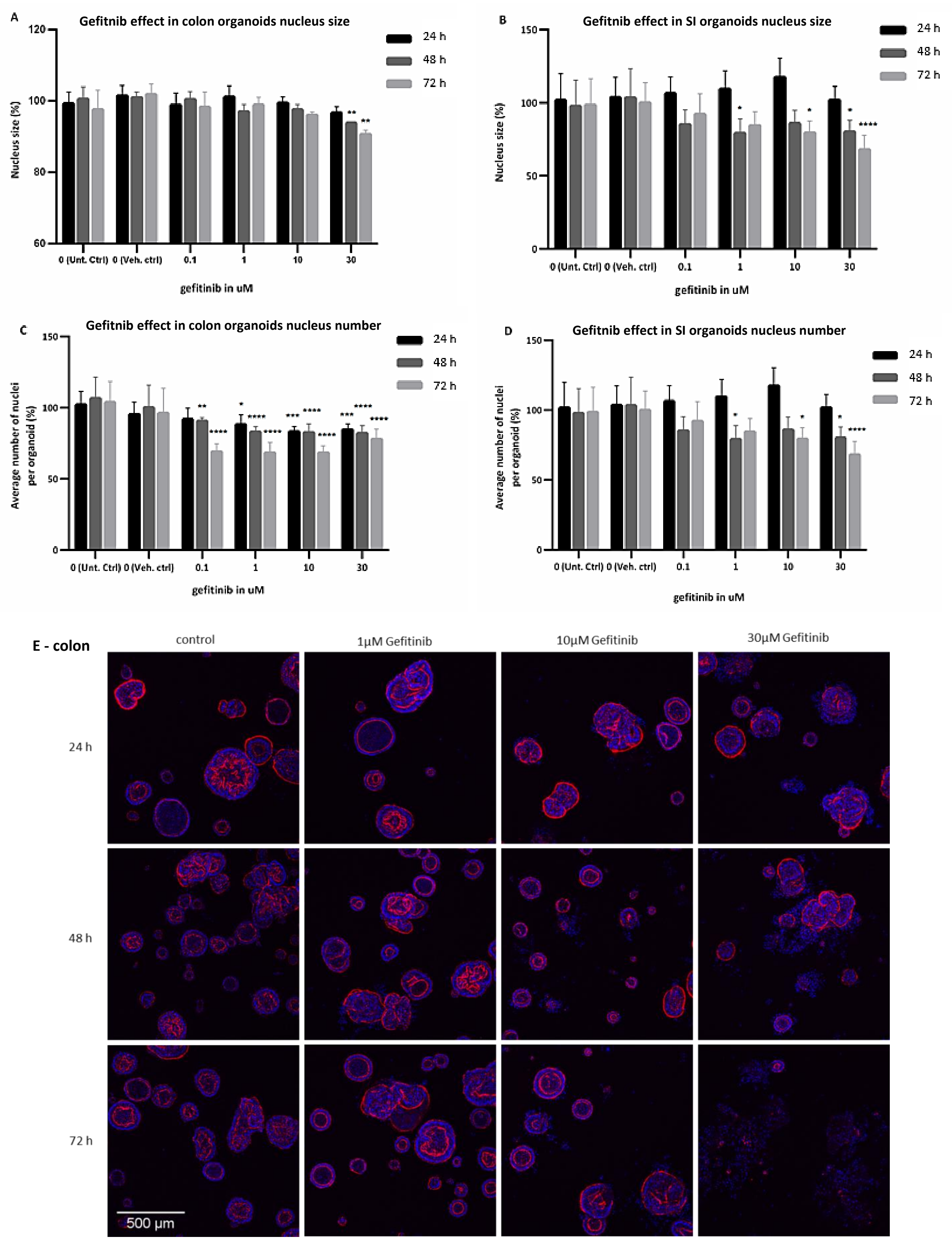
A
Gefitnib effect in colon organoids nucleus size
Nucleus size (%)
0 (Unt. Ctrl) 0 (Veh. ctrl) 0.1 1 10 30
gefitinib in uM
24 h
48 h
72 h
B
Gefitnib effect in SI organoids nucleus size
Nucleus size (%)
0 (Unt. Ctrl) 0 (Veh. ctrl) 0.1 1 10 30
gefitinib in uM
C
Gefitnib effect in colon organoids nucleus number
Average number of nuclei per organoid (%)
0 (Unt. Ctrl) 0 (Veh. ctrl) 0.1 1 10 30
gefitinib in uM
D
Gefitnib effect in SI organoids nucleus number
Average number of nuclei per organoid (%)
0 (Unt. Ctrl) 0 (Veh. ctrl) 0.1 1 10 30
gefitinib in uM
E - colon
control
1μM Gefitinib
10μM Gefitinib
30μM Gefitinib
24 h
48 h
72 h
500 μm

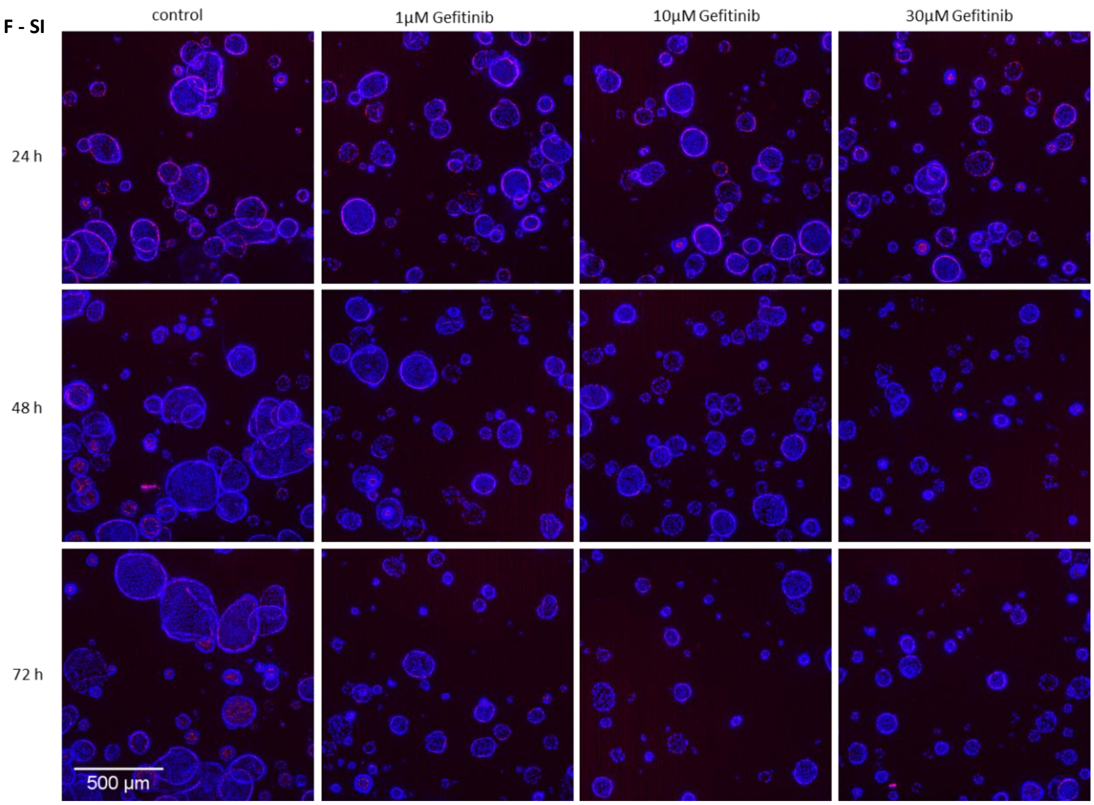

Figure S1. Morphological changes assesses through imaging analysis of healthy colon (A, C and E) and SI (B, D and F) organoids when exposed to 0.1, 1, 10 and 30 µM gefitinib for 24 h in black, 48 h in light grey and 72 h in dark grey, compared with Untreated controls. Values are in % based on fluorescent intensity for each measured parameter. SD was calculated for each condition. Staining in control and treated wells: Rhodamine-phalloidin (actin, in red) and Hoechst (DAPI channel, nuclei, in blue). Legend: Ctrl, control; SD, standard deviation; SI, small intestine; Unt, untreated; Veh, vehicle. *p-value=0.01; **p-value=0.008; ***p-value=0.0008; ****p-value=0.0001.

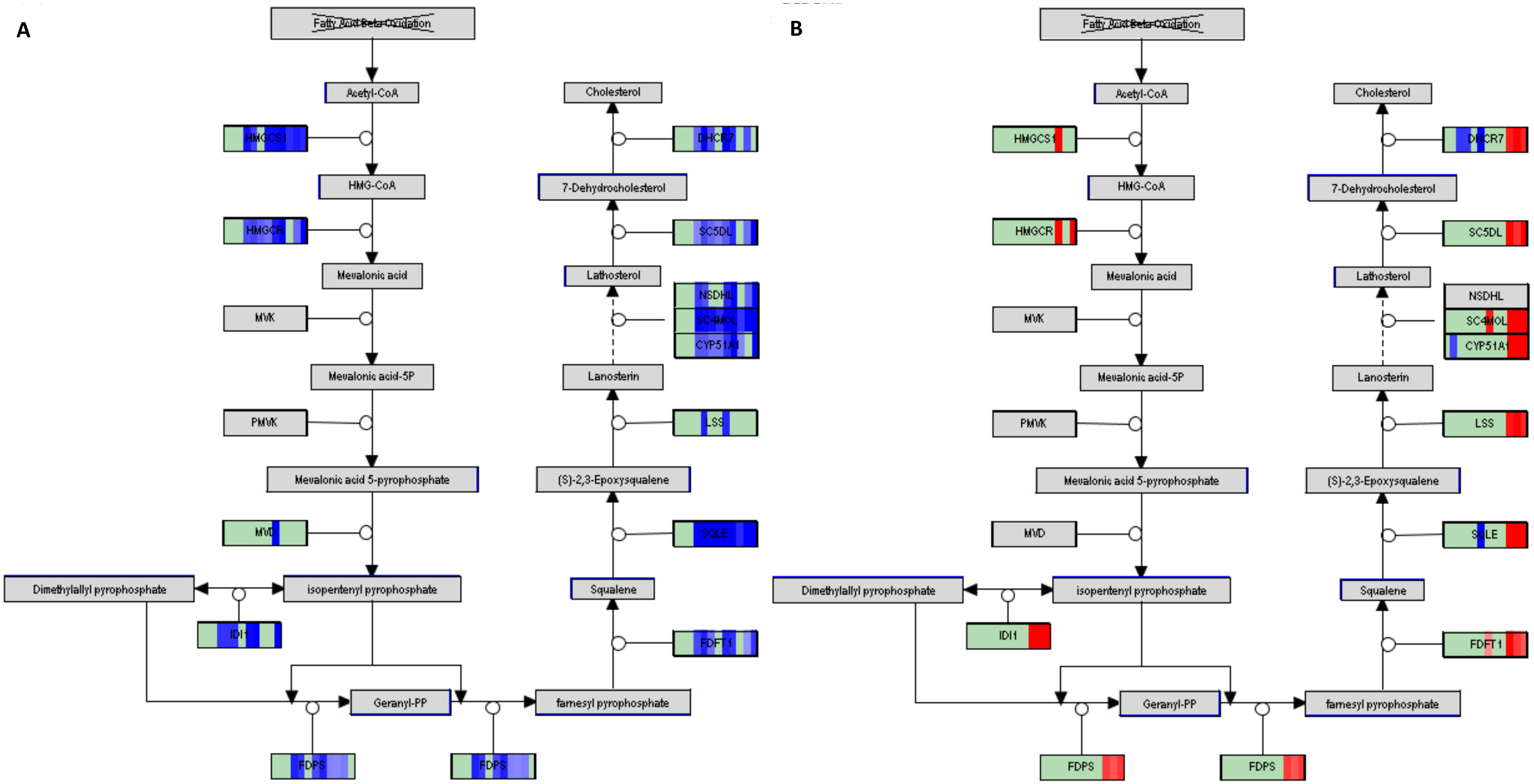

Figure S2. Pathway representation of Cholesterol biosynthesis created with PathVisio based on transcriptomic data obtained from colon (A) and SI (B) organoids considering all treatment conditions with gefitinib. Blue colour represents downregulation and red colour represents upregulation. Colour gradient is based on the DEGs log2FC after Bonferroni correction and indicates the strength of modulation.

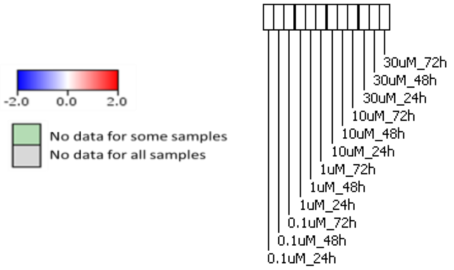

Supplement: Supplementary file 1 [file ijms-23-02213-s001.zip › ijms-1602279-supplementary.pdf]
